# Supplementary material for: Transcriptional divergence of the zebrafish sox17 lineage begins during gastrulation
Source: bioRxiv. 2026 Jun 29:2026.06.27.734843. Preprint. [Version 1] doi: 10.64898/2026.06.27.734843 (PMC13344959; doi:10.64898/2026.06.27.734843)
Supplement: Supplement 2 [file NIHPP2026.06.27.734843v1-supplement-2.pdf]

bioRxiv preprint doi: https://doi.org/10.64898/2026.06.27.734843; this version posted June 29, 2026. The copyright holder for this preprint (which was not certified by peer review) is the author/funder, who has granted bioRxiv a license to display the preprint in perpetuity. It is made available under a CC-BY 4.0 International license.

SUPPLEMENTAL FIGURE LEGENDS

Figure S1. Absolute transcript quantification during early zebrafish development. Absolute transcript abundance of *cdx4, eomes, met, otx1, sox17, sox32,* and *tead1b* was measured by quantitative PCR in whole embryos lysates from 0 to 12 hpf relative to an external standard (Ligunas and Materna, 2026). Maternal transcripts (*eomes* and *otx1*) declined rapidly and were largely undetectable before the onset of gastrulation, whereas transcripts associated with endoderm specification, migration, and regionalization (*sox32, sox17, met, tead1b,* and *cdx4*) were activated prior or during gastrulation. Transcript abundance is reported as transcripts per embryo. Circles and triangles represent independent biological replicates; lines indicate the mean.

Figure S2. Feature plots of marker gene expression used for cluster annotation. UMAP feature plots showing expression of representative marker genes used to assign identities to the major cell populations captured in the *Tg(sox17:GFP)* dataset. Expression levels are overlaid on the integrated UMAP shown in Fig. 2. Color intensity indicates normalized transcript abundance.

Figure S3. Cluster-defining gene expression. Heatmaps showing the top 10 differentially expressed genes for each cluster identified at 8, 10, and 12 hpf, as well as the integrated dataset. Genes were ranked by differential expression relative to all other clusters. Color intensity indicates scaled expression levels.

Figure S4. Fully annotated UMAPs for individual developmental stages. UMAPs showing all annotated cell populations identified at 8, 10, and 12 hpf. In contrast to the simplified annotations shown in Fig. 3, all cluster identities are displayed for each developmental stage. Colors correspond to cluster identities shown in Fig. 2.

bioRxiv preprint doi: https://doi.org/10.64898/2026.06.27.734843; this version posted June 29, 2026. The copyright holder for this preprint (which was not certified by peer review) is the author/funder, who has granted bioRxiv a license to display the preprint in perpetuity. It is made available under a CC-BY 4.0 International license.

Figure S5. Differential gene expression between endoderm and dorsal forerunner cells. Volcano plots showing differential gene expression between endoderm and dorsal forerunner cells at 8, 10, and 12 hpf. The x-axis indicates log2 fold change, and the y-axis shows the adjusted *P* value (−log10-transformed). Genes exhibiting a ≥2-fold difference in expression (|log2 fold change| ≥ 1.3) are highlighted and annotated.

Figure S6. Feature plots of genes enriched in the sox17 lineage. UMAP feature plots showing the expression patterns of genes enriched in endoderm and dorsal forerunner cells relative to the remaining dataset across all developmental stages. Expression levels are overlaid on the UMAPs corresponding to the 8, 10, and 12 hpf datasets, with color intensity indicating normalized transcript abundance.

Figure S7. Differentially expressed genes defining endoderm subclusters. Heatmaps showing genes differentially expressed among endoderm subclusters identified at 8 hpf, 10 hpf, and 12 hpf. These heatmaps correspond to the subclustering analyses shown in Figs. 5 and 7 and highlight the transcriptional programs underlying regional diversification of the endoderm. Genes were ranked by differential expression relative to the remaining endoderm subclusters at each developmental stage. Color intensity indicates scaled expression levels.

bioRxiv preprint doi: https://doi.org/10.64898/2026.06.27.734843; this version posted June 29, 2026. The copyright holder for this preprint (which was not certified by peer review) is the author/funder, who has granted bioRxiv a license to display the preprint in perpetuity. It is made available under a CC-BY 4.0 International license.

Table S1: Sequence of qPCR primers used for time course measurements in Fig. S1. All sequences shown in 5'→3' orientation.

**cdx4-F**	AGTCGTCATCAACCGGCAAA
**cdx4-R**	AAGCCCGAGGTTTACAGCAA
**eomesa-F**	CACCAGAAACTCAACGCACG
**eomesa-R**	TCGGAGGATGAGGACAGGTT
**met-F**	TGTGGACTTGTAGCTCGTGT
**met-R**	GAGCGGAGGAAGGAGAGATC
**otx1-F**	CAGCAACACCGAGAAAGCAG
**otx1-R**	AGTGCCACCTCCTCTCTCAT
**sox17-F**	TTCCTGGGATGTGTGATGCC
**sox17-R**	CCATAGGAGAGGGGTCAGGT
**sox32-F**	ACCAGCTTGATCGCAGTGAA
**sox32-R**	CTGGATGGAAGCAGCAGTCA
**tead1b-F**	ATGGCCGTTTTGTGTACCGA
**tead1b-R**	GTTCTCCAGGACGCTGTTCA
